# Targeting the Replication Initiator of the Second Vibrio Chromosome: Towards Generation of *Vibrionaceae*-Specific Antimicrobial Agents

**DOI:** 10.1371/journal.ppat.1000663

**Published:** 2009-11-20

**Authors:** Yoshiharu Yamaichi, Stéphane Duigou, Elizabeth A. Shakhnovich, Matthew K. Waldor

**Affiliations:** Channing Laboratory, Brigham and Women's Hospital, Harvard Medical School and Howard Hughes Medical Institute, Boston, Massachusetts, United States of America; Yale University School of Medicine, United States of America

## Abstract

The *Vibrionaceae* is comprised of numerous aquatic species and includes several human pathogens, such as *Vibrio cholerae*, the cause of cholera. All organisms in this family have two chromosomes, and replication of the smaller one depends on *rctB*, a gene that is restricted to the *Vibrionaceae*. Given the increasing prevalence of multi-drug resistance in pathogenic vibrios, there is a need for new targets and drugs to combat these pathogens. Here, we carried out a high throughput cell-based screen to find small molecule inhibitors of RctB. We identified a compound that blocked growth of an *E. coli* strain bearing an *rctB*-dependent plasmid but did not influence growth of *E. coli* lacking this plasmid. This compound, designated vibrepin, had potent cidal activity against *V. cholerae* and inhibited the growth of all vibrio species tested. Vibrepin blocked RctB *oriCII* unwinding, apparently by promoting formation of large non-functional RctB complexes. Although vibrepin also appears to have targets other than RctB, our findings suggest that RctB is an attractive target for generation of novel antibiotics that only block growth of vibrios. Vibrio-specific agents, unlike antibiotics currently used in clinical practice, will not engender resistance in the normal human flora or in non-vibrio environmental microorganisms.

## Author Summary

Multi-drug resistant bacteria continue to emerge and there is a pressing need for the development of new antibiotics. Here, we carried out a cell-based high throughput screen to identify inhibitors of RctB, the initiator of replication of the second chromosome found in all the species of the *Vibrionaceae*. This family of bacteria includes several human pathogens, including *Vibrio cholerae*, the cause of cholera, as well as several species that damage economically important marine organisms. We identified a compound—designated vibrepin—that has potent cidal activity against *V. cholerae* and inhibited growth of all vibrio species tested. Vibrepin blocked RctB unwinding of the origin of replication of the second *V. cholerae* chromosome, apparently by promoting the formation of large non-functional RctB complexes. Vibrepin represents a new class of antibiotic that specifically targets a particular family of microorganisms (the *Vibrionaceae*). Such targeted agents will not engender resistance in the normal human flora or in non-vibrio environmental microorganisms. Thus, in principle, genes mediating resistance to these compounds will not arise in and be transferred from non-vibrios to vibrios, perhaps postponing the development of resistance.

## Introduction

The *Vibrionaceae* are a diverse family of bacteria that includes more than 80 species [1],[2]. Vibrios are gram-negative rods that usually inhabit aquatic habitats, often in association with eukaryotes. This large family includes important human pathogens such as *V. cholerae*, *V. parahaemolyticus* and *V. vulnificus*, which can cause gastrointestinal disorders and other illnesses [2],[3]. Vibrio species are also pathogenic for several economically important marine organisms; for example farmed shrimp are harmed by *V. harveyi* and *V. nigripulchritudo* [2],[4],[5].

One notable attribute of the *Vibrionaceae* is their bipartite genomes. The genomes of most other γ-proteobacteria (such as *E. coli*) consist of single circular chromosomes, whereas the genomes of vibrios are comprised of two circular chromosomes [6]. Studies of *V. cholerae*, the agent of cholera, have revealed that different proteins initiate replication of its two chromosomes. Initiator proteins bind to and melt origins of replication and also recruit components of the replisome to the origin [7]. DnaA, a conserved AAA+ ATPase, is thought to be the initiator of chromosome DNA replication in most eubacteria [8],[9], and several observations support the idea that DnaA serves as the initiator of replication of the large *V. cholerae* chromosome (chrI) as well. The origin of replication of chrI, *oriCI*, is similar in sequence to *oriC*, the origin of replication of the *E. coli* chromosome [10], and both contain several binding sites for DnaA. Replication of *oriCI*-dependent minichromosomes is DnaA-dependent [10], and overexpression of DnaA leads to overinitiation of *oriCI* [11]. Finally, DnaA can unwind *oriCI* but can't unwind *oriCII*, the origin of replication of the small *V. cholerae* chromosome (chrII) [12].

Several observations suggest that RctB, a 658 amino acid protein that lacks any known motifs or similarity to characterized initiators, is the initiator of chrII replication. First, RctB binds to several sites within *oriCII* [10]. Second, overproduction of RctB in *V. cholerae* promotes overinitiation of chrII and not chrI [11]. Third, RctB is necessary and sufficient to enable replication of *oriCII*-based minichromosomes in *E. coli*, and the copy number of such minichromosomes increases as the level of RctB is raised [12],[13]. Finally, RctB can unwind *oriCII* and not *oriCI* [12].

*rctB* homologues are encoded by all vibrios that have been tested but are not found outside the *Vibrionaceae* [10]. As a conserved and essential gene product restricted to the *Vibrionaceae*, RctB might be an attractive target for new vibrio-specific antibiotics. Given the increasing prevalence of multi drug resistance in pathogenic vibrios [14]–[18], there is a growing need for new drugs to combat these organisms. Here, we carried out a high throughput cell-based screen to find small molecule inhibitors of RctB. We identified a compound that blocks growth of an *E. coli* strain bearing an *rctB*-dependent plasmid but does not influence growth of *E. coli* lacking this plasmid. This compound, designated vibrepin, also has potent cidal activity against *V. cholerae* and inhibits growth of all vibrio species tested. Genetic and biochemical evidence strongly suggest that this compound, designated vibrepin, targets RctB. Our findings suggest that RctB is a useful drug target for the creation of *Vibrionaceae*-specific antimicrobial agents.

## Results

### A candidate RctB inhibitor identified with a high throughput cell-based screen

**Figure 1:**
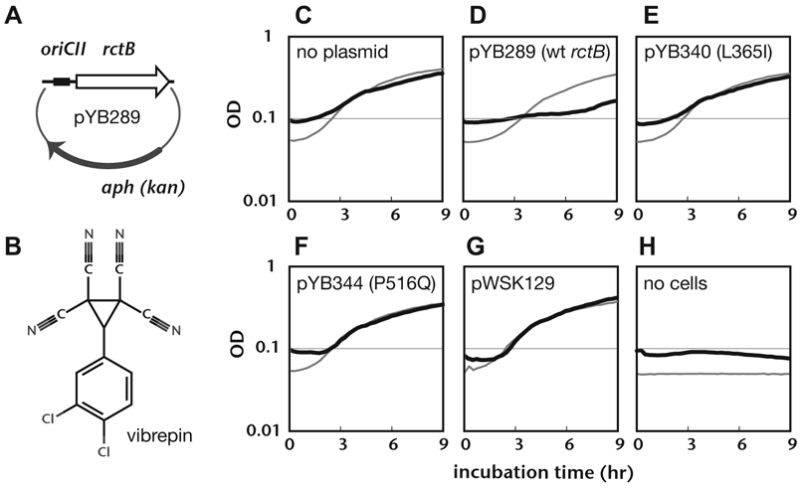
Identification of a small molecule that inhibits RctB-dependent replication. A) Schematic of the RctB-dependent *oriCII*-bearing plasmid, pYB289, used in the high throughput screen for small molecule inhibitors of RctB. B) Structure of vibrepin, one of the compounds identified in the screen. C–G) growth curves of *E. coli* DH5α harboring no plasmid, pYB289 (encoding wt RctB), pYB340 (encoding RctB[L365I]), pYB344 (encoding RctB [P516Q]) and pWSK129 (a non-*oriCII*-based plasmid). Thick black lines represent growth in the presence of vibrepin (16 µg/ml) and gray lines represent growth in the presence of DMSO. Representative growth curves (average of triplicate wells from a plate reader) from 3 or more independent experiments are presented. The higher initial optical density of the cultures containing vibrepin is due to the incomplete solubility of this compound in LB media at 16 µg/ml. This is apparent in H) which shows the optical density generated by vibrepin (thick line), or DMSO (thin gray line) without the addition of cells.

We developed a high throughput cell-based screen to identify small molecule inhibitors of RctB. This assay relied on pYB289, a small plasmid that contains only *oriCII*, *rctB* and a gene (*aph*) that confers resistance to kanamycin (Figure 1A). This plasmid can replicate in *E. coli*, since expression of *rctB* in *E. coli* is sufficient to enable replication of *oriCII-*dependent plasmids in this heterologous host [12],[13]. *E. coli* harboring pYB289 exhibit kanamycin resistance, and we used kanamycin resistance as a marker of this plasmid's replication in our screen. We screened a library of ∼138,000 small molecules for compounds that inhibited growth of *E. coli* Mach1 harboring pYB289 in the presence of kanamycin but did not inhibit growth of Mach1 without the plasmid. Several candidate RctB inhibitors were identified, and one - 3­ (3,4­dichlorophenyl) cyclopropane ­1,1,2,2 ­tetracarbonitrile (Figure 1B), designated here as vibrepin (for vibrio replication inhibitor)- was selected for further study, since its predicted pharmacologic properties were superior to the others.

### Genetic evidence that vibrepin targets RctB

Vibrepin had no effect on the growth (indicated by increased OD_600 nm_ in cultures) of *E. coli* strain DH5α (Figure 1C). In addition, it did not influence growth in the presence of kanamycin of DH5α harboring pWSK129 or pYB190, plasmids with distinct non-RctB dependent origins of replication (pSC101 and pUC, respectively) that contain *aph* cassettes, and so does not appear to interfere with establishment of kanamycin resistance (Figure 1G and data not shown). However,16 µg/ml vibrepin completely inhibited growth in the presence of kanamycin of DH5α containing the RctB-dependent plasmid pYB289 for 6 hr (Figure 1D). Some growth of this strain was detectable after 6 hr, probably because the drug was no longer present, since these cells did not appear to be resistant to vibrepin if tested in fresh media.

Although vibrepin-resistant DH5α/pYB289 did not arise in these assays, growth of DH5α containing one of several derivatives of pYB289 (pYB340 or pYB344) that encode variants of RctB with single amino acid substitutions (RctB L365I and P516Q, respectively, which were identified in an unrelated study, see materials and methods) was not impaired by vibrepin (Figure 1E and F). These strains grew as well in the presence of vibrepin as did DH5α lacking a plasmid. Since the only differences between strains DH5α/pYB289, DH5α/pYB340 and DH5α/pYB344 are single amino acid differences in RctB, these observations strongly support the idea that vibrepin targets RctB.

**Figure 2:**
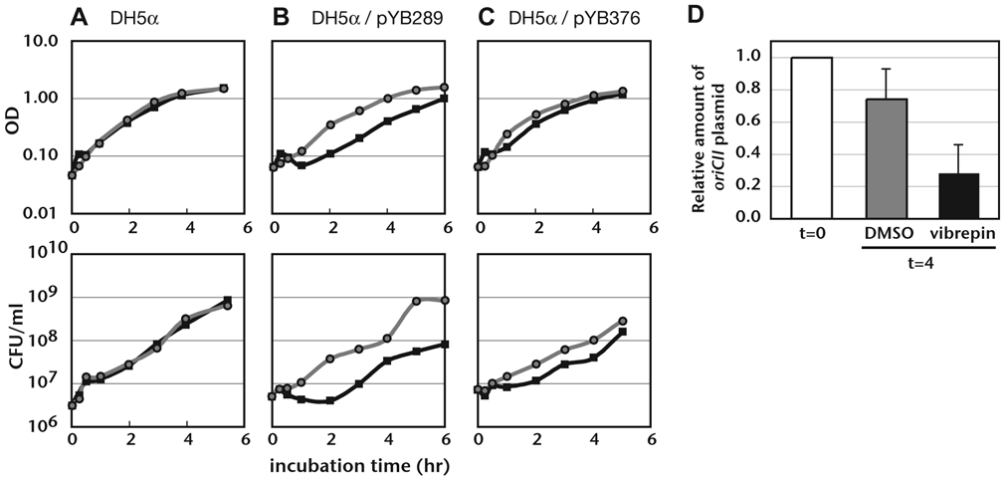
Influence of vibrepin on an *E. coli* strain bearing an RctB-dependent plasmid in the absence of kanamycin. A–C) DH5α (A), DH5α/pYB289 (B), or DH5α/pYB376 (C) were incubated in LB media in test tubes with either vibrepin (16 µg/ml, thick black lines) or DMSO (gray lines). OD_600 nm_ and colony forming units (CFU) were determined at the indicated times. Representative growth curves from 3 or more independent experiments are presented. D) The relative amount of the *oriCII*-based plasmid pYB289 in DH5α after 4 hr of treatment with vibrepin (16 µg/ml) or DMSO. The amount of pYB289 relative to chromosomal DNA was determined before (t = 0) and 4 hrs after treatment (t = 4) using Southern hybridization. The relative amount of pYB289 at t = 0 was set as 1; the mean and standard deviations after 4 hr were calculated from 3 independent experiments.

We also assessed the compound's effect on growth of DH5α/pYB289 in the absence of kanamycin. We anticipated that vibrepin would not inhibit bacterial growth under these conditions, since RctB activity should not be required in the absence of kanamycin. Unexpectedly, we found that vibrepin impaired the growth of DH5α/pYB289 even when plasmid replication was not required. Addition of 16 µg/ml vibrepin to cultures of this strain lacking kanamycin prevented an increase in OD_600 nm_ for ∼2 hr and caused a decrease in the number of viable cells (Figure 2B) but did not influence growth of DH5α lacking this plasmid (Figure 2A). Vibrepin also stimulated the loss of pYB289 from DH5α in the absence of kanamycin selection (Figure 2D). Vibrepin even had a mild inhibitory effect on the growth of DH5α harboring pYB376, a pSC101-based vector containing *rctB* (Figure 2C). Collectively, these results suggest that production of wild type RctB in the presence of vibrepin may have toxic effects, at least in *E. coli*, and that such effects may contribute to the growth inhibition observed in the assay used above. However, since vibrepin was a less potent inhibitor of DH5α/pYB289 growth in the absence of kanamycin than in its presence, it is likely that vibrepin inhibits growth of this strain by more than one mechanism.

### Biochemical evidence that vibrepin targets RctB

**Figure 3:**
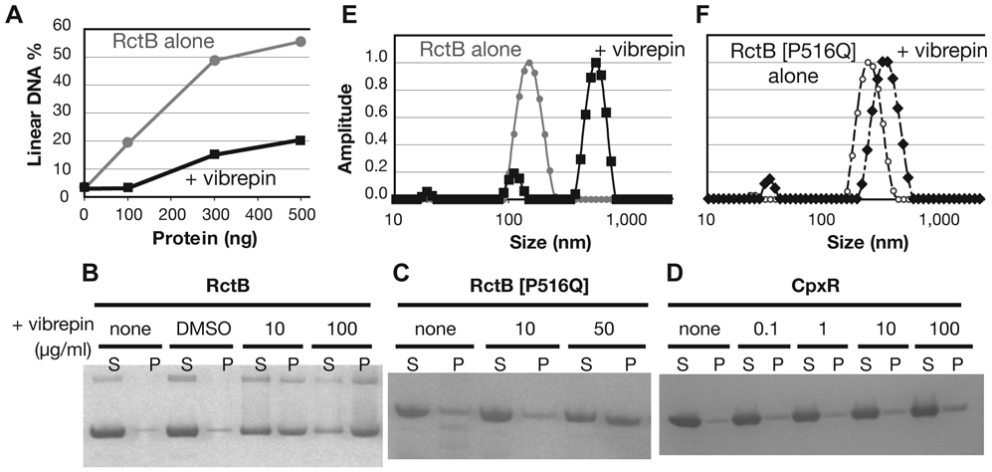
Vibrepin interferes with RctB *oriCII* opening activity and promotes formation of RctB complexes. A) Influence of vibrepin (10 µg/ml) on the *oriCII* unwinding activity of RctB. Representative results from 3 experiments are shown. B–D) Sedimentation of 10 µM of RctB (B), RctB[P516Q] (C) or CpxR (D) alone, with DMSO or with different concentrations of vibrepin. After centrifugation the pellet fraction (P) and the supernatant fraction (S) were loaded on a NuPage SDS gel and stained with Coomassie Blue. E, F) DLS measurements of molecular mass distributions of RctB (E) and RctB[P516Q] (F) preincubated with or without vibrepin; gray circles, RctB alone; black squares, RctB + vibrepin (10 µg/ml); open circles, RctB[P516Q] alone; diamonds, RctB[P516Q] + vibrepin (10 µg/ml).

To assess vibrepin's influence on RctB's activity as a replication initiator, we tested the compound's effects on unwinding of *oriCII* by RctB, using a P1 nuclease-based assay. In this assay, the single-strand specific P1 endonuclease cleaves a plasmid containing *oriCII* if it becomes unwound; linearized plasmid is then detected by agarose gel electrophoresis [12]. As seen in Figure 3A, unwinding of *oriCII* by RctB was markedly inhibited by vibrepin. DMSO, the solvent used to dissolve vibrepin, did not influence RctB unwinding activity (data not shown). These observations are consistent with the hypothesis that vibrepin interferes with RctB function as an initiator of replication by blocking its ability to unwind *oriCII*.

We also explored whether vibrepin influences the oligomeric state of RctB. In our ongoing studies of the mechanism of action of RctB, we found that purified RctB does not pellet after centrifugation at 20,000×*g* for 30 min in the absence of DNA. However, in the presence of 10 µg/ml of vibrepin, ∼50% of RctB was found in the pellet fraction after centrifugation; when higher amounts of vibrepin were added, most of the RctB added to the assay pelleted (Figure 3B). In contrast, RctB [P516Q], which appeared to be resistant to in vivo inhibition by vibrepin (Figure 1F), was less susceptible than wild type RctB to vibrepin-induced aggregation in the pelleting assay (Figure 3C). Furthermore, vibrepin did not promote the aggregation of either CpxR [19] (Figure 3D) or ParA2 (data not shown), DNA-binding proteins unrelated to RctB. Thus, vibrepin does not indiscriminately aggregate proteins. The vibrepin solvent DMSO also did not promote the pelleting of RctB. Together, these findings suggest that vibrepin leads to the formation of high molecular weight complexes of RctB that are no longer soluble. Consistent with these observations, we found that vibrepin increased the apparent radius of RctB complexes approximately 4-fold in dynamic light scattering (DLS) assays (from 153 to 588, Figure 3E). The range of radii of the RctB complexes after addition of vibrepin was narrow, suggesting that vibrepin promotes the formation of RctB complexes of a particular stoichiometry rather than random aggregation of this protein. Although the apparent radius of RctB [P516Q] was greater than that of wild type RctB, vibrepin only had a minor effect (from 257 to 370, Figure 3F). Collectively these observations suggest that vibrepin may interfere with RctB *oriCII* unwinding by promoting the formation of non-functional RctB complexes.

### Diverse vibrios are susceptible to vibrepin

**Figure 4:**
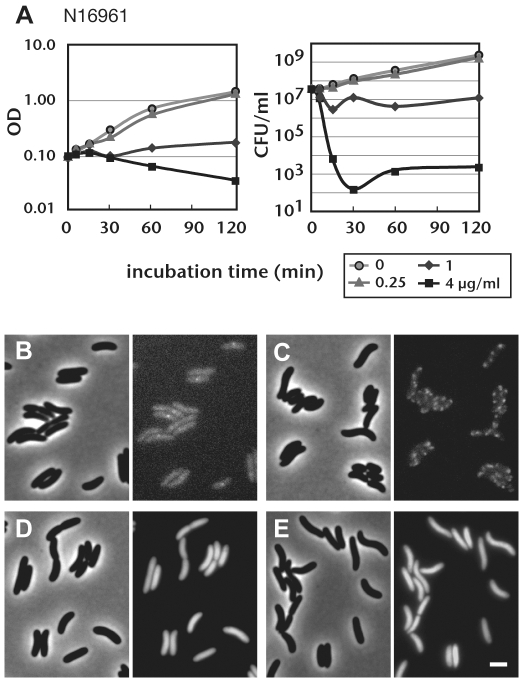
Influence of vibrepin on *V. cholera*e. A) The optical density or number of colony forming units in cultures of the *V. cholerae* N16961 with the indicated concentrations of vibrepin. Representative results from at least 3 independent experiments are shown. B–E) Vibrepin induces aggregation of RctB *in vivo*. Representative fields of phase contrast (left) and GFP signal (right) of *V. cholerae* cells expressing RctB-GFP (B and C) or untagged GFP (D and E) are shown. (B and D) no treatment; (C and E) 1 µg of vibrepin was added 1 hr prior to the imaging. Bar = 2 µm.

RctB is required for replication of *V. cholerae* chrII and is hypothesized to govern chrII replication initiation in all other vibrio species as well, since it is highly conserved. We therefore assessed whether vibrepin could inhibit the growth of vibrio species. Vibrepin prevented growth of *V. cholerae* at doses as low as 1.0 µg/ml (Figure 4A). Used at this concentration, vibrepin induced stasis; however, at higher doses vibrepin had cidal activity. At concentrations of 4 µg/ml, vibrepin reduced the numbers of N16961 colony forming units (CFU) by more the 5 orders of magnitude within 30 minutes (Figure 4A).

Vibrepin also prevented growth of all additional vibrio species tested (Table 1). Drug concentrations ranging from 0.4 to 0.8 µg/ml were sufficient to inhibit the growth of the three major human vibrio pathogens *V. cholerae*, *V. parahaemolyticus* and *V. vulnificus*, as well as the shrimp pathogen *V. nigripulchritudo* (Table 1). In contrast, vibrepin concentrations <13 µg/ml did not inhibit growth of any of the *E. coli* strains we tested, including several pathogenic strains, and most *E. coli* strains were resistant to at least 16 µg/ml of vibrepin (Table 1). Together these observations are consistent with the idea that vibrepin targets RctB, a vibrio-specific essential protein. However, we also observed that vibrepin inhibited growth of *Bacillus subtilis* and *Staphylococcus aureus* (Table 1), two Gram-positive species that lack RctB homologues. Thus, it appears that some organisms contain vibrepin targets other than RctB. The vibrepin target(s) in these Gram-positive bacteria has yet to be defined.

**Table 1:** Minimal inhibitory concentrations (µg/ml)^1^ of vibrepin for various bacteria.

*V. cholerae* N16961	1.5
*V. fluvialis* H-08942	2.0
*V. parahemolyticus VP47*	0.7
*V. vulnificus* ATCC 27562	0.4
*V. nigripulchritudo*	0.8
*E. coli* DH5α	>16
*E. coli* MG1655	>16
EPEC 2348/69	>16
EHEC EDL933	13
*B. subtilis* PY79	1.0
*S. aureus* MW2	2.0

1 Concentrations of vibrepin that blocked increases in OD_600 nm_ for at least 6 hours. The average values derived from at least 3 experiments are shown. When 16 µg/ml did not inhibit growth, a value of >16 is shown.

Several approaches were taken to confirm the target of vibrepin in *V. cholerae*. First, we repeatedly screened for *V. cholerae* mutants that had spontaneously acquired resistance to vibrepin; however, resistant colonies were never obtained. We were not able to introduce point mutations in the chromosomal copy of *rctB*. However, we transformed *V. cholerae* with plasmids encoding alleles of RctB that were resistant to vibrepin in *E. coli* (pYB303 and pYB345, encoding RctB L365I and P516Q, respectively), and assessed whether they conferred resistance. Exogenous production of RctB from these plasmids (which did not alter *V. cholerae*'s growth rate) did not render cells resistant (data not shown), suggesting that the presence of wt RctB results in dominant sensitivity, as might be expected given viprepin's toxicity in DH5α/pYB289 even in the absence of plasmid selection. Alternatively, *V. cholerae* may contain vibrepin targets in addition to RctB, which confer sensitivity even in the presence of resistant *rctB* alleles. Such targets may be related to the non-RctB targets that must exist in Gram-positive organisms.

### Vibrepin promotes RctB aggregation *in vivo*

We used an RctB-GFP fusion protein to explore whether vibrepin altered the subcellular distribution of RctB in *V. cholerae*. We constructed a strain where *rctB-gfp* is expressed from the native *rctB* promoter by introducing a gene encoding GFP in-frame at the 3′ end of *rctB*. Since this *rctB-gfp* fusion is the only copy of *rctB* in the cell and this strain had wild type growth (data not shown), the RctB-GFP fusion protein must be functional. In this strain, the distribution of RctB-GFP was generally diffuse, though small foci, usually near mid cell, were occasionally observed (Figure 4B). One hour after addition of 1 µg/ml of vibrepin, the diffuse pattern of RctB-GFP fluorescence was no longer observed; instead, large puncta of RctB-GFP were seen (Figure 4C). In control experiments, we found that vibrepin treatment did not alter the pattern of untagged GFP fluorescence (Figure 4D and E), consistent with our observation that vibrepin does not lead to indiscriminate aggregation of proteins in vitro. These data suggest that the RctB complexes induced by vibrepin in vitro may be a reflection of its mode of action *in vivo*, and that vibrepin's toxicity for *V. cholerae* may result, at least in part, from induction of RctB aggregation.

### Comparative analysis of vibrepin and a structural analog

**Figure 5:**
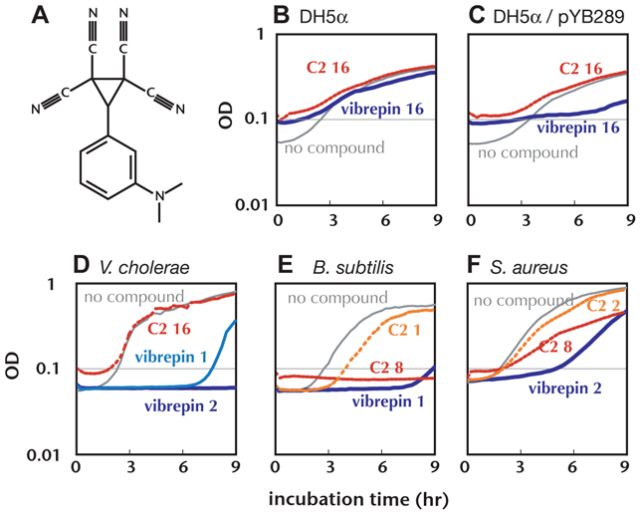
Compound C2 does not inhibit growth of *V. cholerae* but C2 and vibrepin inhibit *B. subtilis* and *S. aureus* growth. A) Structure of compound C2. B–F) Growth curves of the indicated strains with addition of the indicated concentrations (µg/ml) of vibrepin (blue line), C2 (red line) or DMSO control (gray line). Representative growth curves (average of triplicate wells) from 3 or more independent experiments are presented.

Different vibrepin targets could recognize distinct moieties in this compound. In an initial structure activity study of vibrepin, we identified a compound [3-(3-dimethylamino-phenyl)-cyclopropane-1,1,2,2-tetracarbonitrile] (referred to as C2), that is similar in structure to vibrepin and that contains the same highly substituted cyclopropane moiety linked to a phenyl group (compare Figures 5A and 1B). However, C2 did not inhibit growth of DH5α/pYB289 (Figure 5C) or *V. cholerae* (Figure 5D). Since C2 lacks the chlorine substitutions on the phenyl group that are present in vibrepin, these observations may suggest that the chlorines in vibrepin are important for its targeting/inhibition of RctB. However, C2 is not void of antibiotic activity. This compound inhibited growth of *B. subtilis* (Figure 5E) and *S. aureus* (Figure 5F), albeit with lower potency than vibrepin. Together, these observations raise the possibility that the additional (non-RctB) targets of vibrepin may interact with moieties of this compound that are chemically distinguishable from the parts of the molecule that inhibit RctB.

## Discussion

The development of new agents to combat emerging multidrug resistant pathogens is a critical challenge for infectious disease research [20],[21]. In recent years, multidrug resistance in *V. cholerae, V. vulnificus*, and *V. parahaemolyticus*, important human pathogens [14]–[18], and in vibrio species that damage shellfish and other marine organisms raised in aquaculture facilities [22] has been reported. Since RctB is required for replication of the vibrio second chromosome and conserved among the *Vibrionaceae*, we reasoned that RctB could be a target for development of antibiotics that specifically target vibrios. Our cell-based screen for small molecules that inhibited growth of an *E. coli* strain containing an *oriCII*- and RctB-dependent plasmid yielded vibrepin. Evidence that this compound targets RctB includes the observations that vibrepin did not inhibit growth of *E. coli* lacking the RctB-dependent plasmid or *E. coli* strains bearing nearly identical *oriCII*-dependent plasmids that contained single amino acid substitutions in RctB. Furthermore, vibrepin blocked unwinding of *oriCII* by RctB, apparently by promoting the formation of non-functional RctB complexes. Finally, vibrepin inhibited the growth of all vibrio species tested and had potent cidal activity against *V. cholerae*. Although vibrepin also appears to have targets other than RctB, our findings suggest that RctB is an attractive target for generation of antibiotics that only block growth of vibrios.

One mechanism by which vibrepin appears to inhibit RctB function is by promoting formation of RctB complexes. Vibrepin induction of RctB complexes is relatively specific, as the compound hardly affected the oligomeric state of RctB[P516Q] and did not induce multimerization of two DNA-binding proteins unrelated to RctB, CpxR and ParA2. There may be several consequences of RctB aggregation. First, since vibrepin inhibited RctB-mediated unwinding of *oriCII*, RctB complexes may be unable to initiate chrII replication. Second, since vibrepin impaired growth of DH5α/pYB289 in the absence of kanamycin, formation of RctB complexes may be toxic to cells even when RctB-dependent replication is not required. To date, the mechanism underlying such toxicity is unknown. Vibrepin also has targets other than RctB, since this compound inhibited the growth of bacterial species that do not encode RctB orthologues. The multiple effects of vibrepin on RctB as well as the possible existence of a non-RctB vibrepin target(s) in *V. cholerae* likely explains our inability to isolate vibrepin-resistant *V. cholerae* mutants.

Bacteria-specific mediators of DNA replication might be expected to be attractive targets for antimicrobial agents. Recently, O'Donnell and colleagues identified a small molecule that inhibits the interaction of the *E. coli* β-clamp with DNA polymerases using an in vitro biochemical screen [23]. This compound did not inhibit the interaction of the yeast clamp with polymerase and hence should not target eukaryotes; however, it is not known whether this compound inhibits bacterial growth. Inhibitors of DnaA, the initiator of chromosome DNA replication in almost all eubacteria, might also have potential as broad spectrum antibiotics and Skarstad and colleagues reported the development of a high throughput cell-based assay to identify such inhibitors [24]. However, no antibiotics are currently in use that directly target bacterial chromosome replication.

We found that RctB activity can be inhibited and thus showed that this initiator of replication of the second vibrio chromosome is a reasonable target for development of new Vibrionaceae-specific antimicrobial agents. Even though vibrepin has targets besides RctB, we anticipate that it will be possible to modify vibrepin or find new compounds that only target RctB. All antibiotics used in the clinic today are relatively broad spectrum and target highly conserved cellular processes. Therefore, these compounds inevitably select for resistant organisms in the normal human flora as well as in environmental microorganisms after antibiotics are shed into the environment. Genes that confer resistance can then be horizontally transmitted from bystander bacteria to pathogens. Vibrionaceae-specific antibiotics will not engender resistance in the normal human flora or in non-vibrio environmental microorganisms. Thus, in principle, genes mediating resistance to these compounds will not arise in and be transferred from non-vibrios to vibrios, perhaps postponing the development of resistance. Vibrio–specific agents may be useful as new agents for aquaculture and in the prevention and treatment of human vibrioses.

## Materials and Methods

### High throughput screen for RctB inhibitors

High throughput screening for small molecule inhibitors of growth of YBA685 in the presence of kanamycin was carried out at the NSRB screening facility at Harvard Medical School. YBA685 is *E. coli* strain Mach1 containing pYB289, an *rctB*-dependent vector (Figure 1A). Mach1 (Invitrogen), which grows faster than most laboratory *E. coli* strains, was used for the screening phase of our study to minimize the time required to detect growth inhibition. For screening, an overnight culture of YBA685 was inoculated at a 1∶500 dilution into LB broth containing kanamycin (50 µg/ml); the culture was grown at 37°C until reaching an OD_600 nm_ of ∼0.1; then, 30 µl aliquots of the culture were transferred into 384-well plates. The compound library (100 nl of 5 mg/ml in DMSO, final concentration 16.7 µg/ml) was pin-transferred to the plates in duplicate. After the plates were incubated at 37°C for 3 hours, the OD_600 nm_ of the wells was measured. We used *Z*-scores [25] to evaluate the growth inhibition of the compounds tested. The mean and standard deviation of the OD_600 nm_ values from the experimental wells in each plate were obtained; then *Z*-scores for each well were calculated as the difference between the OD_600 nm_ values in each well and the average OD_600 nm_ divided by the standard deviation. Compounds with *Z*-scores below −3 in duplicate plates were considered positive hits. There were 149 positive hits among 137,694 compounds screened. These compounds were counter-screened to exclude molecules that inhibited the growth of Mach1 in the absence of pYB289. Ultimately, we identified four compounds that inhibited growth of YBA685 in the presence of kanamycin but did not inhibit Mach1 growth. Vibrepin (CID 2803695) and C2 were purchased in milligram quantities from Maybridge (Tintagel, UK) and Sigma-Aldrich (St. Louis, MO), respectively. All compounds were dissolved at 50 mg/ml concentration in DMSO and stored at −20°C.

### Strains and plasmids

The strains and plasmids used in this study are listed in Table 2 and 3, respectively.

**Table 2:** Strains used in this study.

Strain	Relevant characteristics	Sources/References
mach1		invitrogen
YBA601	DH5α/pYB289	This study
YBA685	mach1/pYB289	This study
YBA796	DH5α/pYB340	This study
YBA803	DH5α/pYB344	This study
EHEC EDL933		[27]
EPEC 2348/69		[28]
N16961		[29]
YBB182	N16961 *lacZ::gfp*	This study
YBB697	N16961/pYB303	This study
YBB815	N16961/pYB345	This study
YBB874	N16961 *rctB*-gfp	This study
*V. nigripulchritudo*		[5]
*V. parahaemolyticus* VP47	O3:K6 clone	[30]
*V. vulnificus* ATCC 27562		[31]
*V. fluvialis* H-08942		[32]
*B. subtilis* PY79		[33]
*S. aureus* MW2		[34]

**Table 3:** Plasmids used in this study.

Plasmid	Relevant characteristics	Sources/References
A-52	*rctB L365I oriCII rctA aph*	This study
A-57	*rctB P516Q oriCII rctA aph*	This study
pCVD442	Allele exchange vector, *Amp^R^ sacB mob^+^*	[35]
pET28b	IPTG inducible expression vector	Novagen
pET-RctB	pET28b *rctB*	[12]
pGZ119EH	IPTG inducible expression vector	[36]
pJZ111	pCVD442 derivative to construct *lacZ::gfp*	[37]
pOriII	pBR332 *oriCII*	[12]
pWSK129	pSC101 *ori aph*	[38]
pYB190	pUC *ori aph*	[26]
pYB289	*rctB oriCII aph*	[12]
pYB303	pGZ119 *rctB* [L365I]	This study
pYB340	*rctB L365I oriCII aph*	This study
pYB344	*rctB P516Q oriCII aph*	This study
pYB345	pGZ119 *rctB* [P516Q]	This study
pYB346	pET28b *rctB* [P516Q]	This study
pYB364	pCVD442 derivative to construct *rctB-gfp* chromosomal fusion	This study
pYB376	pWSK129 *rctB*	This study

As part of an on-going project to study *oriCII*-based replication, we have isolated a series of mutant *oriCII*-based plasmids that contain *rctA*, a negative regulator of RctB, and mutations in *rctB* (see Materials and Methods of ref. [12]). These mutant *rctB* alleles can support replication of *oriCII*, at least in *E. coli*. We tested the sensitivity of DH5α harboring several of these mutant oriCII plasmids to vibrepin and found that plasmids A-52 and A-57 containing *rctB*[L365I] and [P516Q] respectively confer resistance to the compound. *rctA* was deleted from A-52 and A-57, yielding pYB340 and pYB344 respectively, using the QuickChange XL Site Directed Mutagenesis Kit (Stratagene). The later 2 plasmids are otherwise identical to pYB289, which contains wild type *rctB*. The copy numbers of pYB289, pYB340 and pYB344 in *E. coli* DH5α were approximately the same and the growth rates of DH5α bearing any of these 3 plasmids were indistinguishable. To create *rctB* [L365I] or *rctB* [P516Q] expression vectors, the relevant *rctB* variant was PCR amplified and then inserted into pGZ119EH as previously described [12]. *rctB* [P516Q] was inserted into pET28b (Novagen) (yielding pYB346), for high level expression of C-terminal His tagged RctB [P516Q]. All the relevant DNA sequences of all vectors used in this study were determined. The sequences of the PCR primers used in this study are available upon request.

A *V. cholerae* strain containing a *rctB-gfp* translational fusion in the *rctB* locus and under the control of the native *rctB* promoter (YBB874) and a strain harboring *gfp* gene inserted in the *lacZ* locus under the control of the *Plac* promoter (YBB182) were constructed by allele exchange techniques using pCVD442-based plasmids (pYB364 and pJZ111, respectively) as described [26].

### Growth curves

A SynergyHT microplate reader (BioTek, Winooski, VT) was used to determine the growth kinetics shown in Figures 1 and 5. In these experiments, overnight cultures were diluted 1∶200 and then incubated for 9 hours. For the growth curves shown in Figures 2 and 4A, overnight cultures were diluted into fresh media in test tubes and OD_600 nm_ and CFU were determined at the indicated time points. All cultures were grown in LB media at 37°C except Tryptic Soy Broth (BD) was used for *S. aureus* cultures and LB containing 0.5 M NaCl was used for *V. nigripulchritudo* cultures which were grown at 30°C.

### Quantification of *oriCII* plasmid

The copy numbers of *oriCII-*based plasmids pYB340 and pYB344 relative to pYB289 were measured by quantitative PCR as described previously [12]. For Figure 2D, Southern hybridization of total genomic DNA was used to quantify pYB289 plasmid DNA (relative to *E. coli* chromosomal DNA) in vibrepin treated and untreated cells. A ∼600 bp DNA fragment of *rctB* was used as a probe for pYB289 and a similar sized *narW* fragment was used as a probe for the *E. coli* chromosome. Probe preparation and detection were carried out with the ECL Direct nucleic acid labeling and detection system (GE Healthcare) according to the manufacture's instructions. Bands were quantified using a Fujifilm FLA-5100 imager.

### P1 nuclease cleavage assay

The P1 nuclease-based assays for RctB unwinding of *oriCII*-containing plasmid substrates were performed as described previously [12]. Briefly, different concentrations of C-terminal His-tagged versions of RctB was mixed with 150 fmol of pOriII in 50 µl of a solution composed of 10 mM Hepes-KOH (pH 7.6), 8 mM magnesium acetate, 30% glycerol and 320 µg/ml BSA. After 10 min at 37°C, 1.2 units of P1 nuclease was added to each reaction for 30 seconds; the reactions were stopped with 40 µl of stop buffer (25 mM EDTA, 1% SDS). For quantification of the linearized fraction of the pOriII substrate DNA, an aliquot of the reaction was electrophoresed on a 0.8% agarose gel and then stained with ethidium bromide; the proportion of the plasmid DNA linearized by P1 nuclease was determined using densitometry.

### Sedimentation assay

Purified C-terminal His-tagged RctB or RctB[P516Q] (10 µM) was incubated in 20 µl buffer F (30 mM Tris pH 8, 5 mM MgSO_4_, 100 mM KCl, 2 mM DTT) in the absence or presence of compounds for 10 min at 30°C. Reactions were then centrifuged for 30 min at 4°C at 20,000×*g* in a refrigerated table top centrifuge. The supernatant and pellet fractions were resuspended in 1X loading dye, electrophoresed on SDS PAGE gels and then the amount of protein in each fraction was determined by coomassie staining.

### Dynamic light scattering

DLS assays with a Viscotek 802 (He–Ne laser, 633 nm) were used to evaluate the effects of vibrepin on the apparent molecular mass of RctB and RctB[P516Q]. To remove any protein aggregates prior to the DLS assays, the proteins were centrifuged for 30 min at 20,000×*g* and then the supernatants were collected and filtered through a 0.1 µm ultrafree MC Milipore filter. In each assay, 300 ng of protein was added to a 12 µl reaction in sedimentation buffer F in a quartz cuvette in presence or absence of compounds. The amplitude plotted on the y-axis in Figure 3E and F is reflective of the intensity measurements generated in these experiments; intensity is proportional to the size and concentration of the scattering particles. Amplitude values were calculated using Omnisize 3.0 software. Each curve is representative of at least 5 measurements.
